# Can thrombocytosis or thrombocytopenia predict complicated clinical course and 30-days mortality in patients with pneumonia?

**DOI:** 10.3906/sag-2010-333

**Published:** 2021-06-12

**Authors:** Raziye Sinem MISIRLIOĞLU, Ersin AKSAY, Emre ŞANCI, Kemal Can TERTEMİZ

**Affiliations:** 1 Department of Emergency Medicine, Antalya Ataturk State Hospital, Antalya, Turkey; 2 Department of Emergency Medicine, Faculty of Medicine, Dokuz Eylul University, İzmir, Turkey; 3 Department of Emergency Medicine, University of Health Sciences, Kocaeli Derince Training and Research Hospital, Kocaeli, Turkey; 4 Department of Pulmonary Medicine, Faculty of Medicine, Dokuz Eylul University, İzmir, Turkey

**Keywords:** Pneumonia, platelet, thrombocytosis, thrombocytopenia, mortality, emergency medicine

## Abstract

**Background/aim:**

While several different scoring systems aim to determine the clinical outcomes for patients with pneumonia, there is limited emphasis on the platelet count. This study investigated the relationships between thrombocyte count and 30-day mortality and complicated clinical course of patients with pneumonia.

**Materials and methods:**

This prospective cross-sectional study enrolled patients over 18 years old with a diagnosis of pneumonia in the emergency department for six months. The primary outcome was to establish the relationship between platelet count, mortality, complicated clinical course, and initial vital parameters on admission. The secondary outcome was comparing the platelet count with mortality and complicated clinical course during the hospital stay.

**Results:**

Four hundred-five patients were included (58.8% male, mean age 75.1 ± 12.7 years). On admission, thrombocytosis was observed in 14.1% and thrombocytopenia in 4.2%. There was no difference between the 30-day mortality according to the platelet count at admission and follow-up. Patients who developed thrombocytopenia during follow-up needed more intensive care admissions, invasive mechanical ventilation, noninvasive mechanical ventilation, and vasopressor treatment, while patients with thrombocytosis needed invasive mechanical ventilation more frequently.

**Conclusion:**

Neither thrombocytopenia nor thrombocytosis is not associated with 30-day mortality in ED patients with pneumonia. Thrombocytopenia during follow-up was associated with a higher incidence for a complicated clinical course.

## 1. Introduction

Several studies aimed to determine the relationship between platelet count and clinical outcome of patients with pneumonia have gradually increased in recent years [1–5]. Most of these studies have reported that thrombocytosis and thrombocytopenia are independent risk factors for mortality. Scoring systems predicting adverse outcomes in patients with pneumonia are in clinical use, such as a PSI (pneumonia severity index), CURB-65, SMART-COP, and CAP-PIRO. However, neither thrombocytopenia nor thrombocytosis is accepted as a risk factor.

Platelets have been shown to play an important role in the formation of immune system responses other than their role on the coagulation system. It has been demonstrated that thrombocytes activate neutrophils and monocytes, secrete pro-inflammatory proteins and antimicrobial peptides, and express several immune-related receptors. Streptococcus pneumonia is known to activate platelets directly [1]. However, platelet count decreases due to bone marrow depression in severe infections and sepsis, associated with increased mortality. Thrombocytopenia is one of the risk factors in SOFA (sequential organ failure assessment) and MEDS (mortality in emergency department sepsis) score in patients with sepsis. Pneumonia is accepted as one of the common etiologic factors for sepsis [2]. However, the platelet count is not a high-risk factor in many scores used in determining risk in patients with pneumonia.

This study aimed to determine the relationship between thrombocyte count with 30-day mortality and the complicated clinical course of patients with pneumonia.

## 2. Materials and methods

The study was conducted as a prospective cross-sectional study in a tertiary academic emergency department (ED) with an annual census of 135,000 patients with many geriatric patients with a severe lower respiratory infection. The patients over 18 years old who presented to the ED with undifferentiated pneumonia (community-acquired pneumonia, nosocomial pneumonia, and healthcare-associated pneumonia) between February to August 2017 were included in the study. The diagnosis of pneumonia had been made with the positive radiologic findings and appropriate clinical presentation and confirmed by the consultant of pulmonary medicine. Patients who had hematologic diseases that can decrease or increase the platelet count (such as aplastic anemia, chronic lymphoblastic leukemia, idiopathic thrombocytopenic purpura) and did not want to participate in the study were excluded. Approval of the Ethics Committee of Dokuz Eylul University had been obtained. (Decision no: 2017/01-33, date: 12.01.2017)

Demographic and clinical variables, laboratory results, radiologic findings, pneumonia severity index grades, CURB-65 scores, adverse outcomes, and mortality rates were collected. The mortality rate of the patients discharged from the hospital within 30 days after ED admission was investigated by telephone survey; if the patient or relatives could not be reached, the national death notification system was used to confirm mortality.

Blood gas analysis was measured with Radiometer ABL800 Basic, hematologic parameters, including platelet count, were measured with Beckman Coulter LH780. Thrombocytopenia was defined as platelet count ≤ 100,000/L. Thrombocytosis was defined as platelet count ≥ 400,000/L.

The primary outcome of the study was the evaluation of the relationship between platelet count on ED admission with 30-days mortality and complicated clinical course. Complicated clinical course had been defined if the patients need at least one of these: noninvasive/invasive mechanical ventilation, intensive care unit admission, or vasopressor treatment. The secondary outcome was the evaluation of the relationship between platelet count on follow-up with 30-days mortality and complicated clinical outcome. During the follow-up, the lowest or highest levels of repeated platelet measurements were evaluated.

### 2.1. Statistical analysis

Statistical analysis was performed using Statistical Package for Social Sciences for Windows v. 24.0. For categorical variables, the number and percentage were expressed as mean and standard deviation for numerical variables. Kolmogorov–Smirnov and Shapiro–Wilk tests were used for the normality analysis. The means of the normally distributed numerical values were compared with a paired t-test, and the means of the nonnormally distributed numerical values were compared using the Wilcoxon test. Chi-square and Fisher exact tests were used to compare categorical variables. Odds ratio, negative and positive predictive values were calculated from “vassarstats.net”. P values < 0.05 were considered statistically significant.

## 3. Results

Pneumonia has been diagnosed in 461 patients during the study period. Fifty-six of these patients were excluded from the study (41 of those had a hematologic disease and 15 patients refused to participate in the study). Four hundred-five patients were enrolled in the study. The mean age was 75.1 ± 12.7 years (min-max 18–97), and 58.8% of patients were male. The majority of the patients had lobar and multilobar infiltrations. Furthermore, most of the patients included in the study were PSI class IV and V. The demographic and clinical features of the patients are presented in Table 1.

### 3.1. The primary outcomes (the relationship between platelet count on admission and mortality, complicated clinical course, and initial vital parameters)

Median platelet count was 245,000/L (interquartile range [IQR] 182.500–335.000/L, min–max 7000–−845.000/L) on ED admission. Thrombocytosis was detected in 14.1% of patients, and thrombocytopenia was detected in 4.2%. The median platelet count was 241,000/L (IQR 184.000–332.000/L, min–max 12000–773.000/L) in patients with 30 days survival, and 273,000 (IQR: 180.000–341.750/L, min–max 7000–845.000/L) for nonsurvivors (p = 0.72). Thrombocytopenic patients were more prone to a higher 30-day mortality rate and had a higher incidence of complicated clinical course. However, there were no significant differences between the patients with normal thrombocyte count and those with thrombocytosis or thrombocytopenia for the rates of 30-day mortality. The rates for ICU admission, the need for invasive mechanical ventilation, the need for vasopressors, and the need for noninvasive mechanical ventilation were similar in groups, except the need for invasive mechanical ventilation was more common in patients with thrombocytopenia (p = 0.037). Thrombocytopenic patients had significantly lower systolic and diastolic blood pressure than those with normal thrombocyte count. The relationships between platelet count on admission and mortality, complicated clinical course, and initial vital parameters are shown in Table 2.

### 3.2. The secondary outcomes (the relationship between platelet count during hospital follow-up and mortality, and complicated clinical course)

Serial complete blood count had been obtained in 245 (60.5%) patients during hospital follow-up; 27.7% of those had developed thrombocytosis, 10.2% had developed thrombocytopenia. Seven patients developed both thrombocytopenia and thrombocytosis in their clinical course.

During follow-up, those patients that developed thrombocytopenia had increased rates of ICU admission (p = 0.036), invasive mechanical ventilation (p = 0.001), noninvasive mechanical ventilation (p = 0.022), and vasopressor treatment (p < 0.001), compared to patients with normal thrombocyte count. Mortality was higher in thrombocytopenic patients (44% vs. 29.6%), but no statistical difference was observed. The need for invasive mechanical ventilation was higher in patients with thrombocytosis (p = 0.035). Associations between platelet count on follow-up and 30-days mortality and complicated clinical course are shown in Table 3.

## 4. Discussion

There was no association between platelet counts and 30-day mortality in patients diagnosed with pneumonia in the emergency department. While there was no correlation between thrombocytosis at the time of admission and the clinical course of complications, thrombocytopenic patients had lower blood pressure at admission to ED, and invasive mechanical ventilation was more frequently used. 30-day mortality, ICU admission, and noninvasive mechanical ventilation were more frequent in patients with thrombocytopenia at presentation; however, there was no significant statistical difference.

Although 30-day mortality, ICU admission, and noninvasive mechanical ventilation were more frequent in patients with thrombocytopenia at presentation, the absence of a statistically significant difference is likely due to the low number of thrombocytopenic patients. ICU admission, invasive and noninvasive mechanical ventilation initiation and vasopressor requirements were statistically higher in patients who developed thrombocytopenia during follow-up.

One of the first studies examining the relationship between platelet level and mortality in pneumonia patients was published in 2007 [3]. The count of thrombocytes below 50 × 10^9^/L in community-acquired pneumonia patients admitted to the intensive care unit was defined as an independent risk factor for mortality (adjusted OR 4.386, 95% CI 2.023–9.511, p = 0.0014).

In 2010, a retrospective study addressed the relationship between thrombocyte count and prognosis. This study included 500 patients hospitalized with pneumonia in a regional army center showing thrombocytosis was found in 75% of the survivors and 25% of the nonsurvivors [4]. (p < 000.1) multivariate logistic regression analysis found that mortality was significantly higher in patients with thrombocytosis (OR, 3.268; 95% CI, 1.578–6.770, p = 0.001). Propensity-adjusted risk of 30-day mortality was approximately 5% in patients’ platelet counts in the range of 150,000 to 250,000 cells/mL. However, this value was 15% in patients with platelet count >400,000 cell/mL. Thrombocytopenia also had been reported to be associated with an increased risk of mortality. In this study, patients with community-acquired pneumonia, even if they had hematologic diseases, have been enrolled in the study. We enrolled all patients with pneumonia in our study regardless of etiologic cause; however, we excluded the patients with hematological diseases.

Prina et al. conducted a multicenter prospective study in 2013, determining that thrombocytosis was an increased risk factor for mortality in 2423 community-acquired pneumonia patients [5]. Patients with immune suppression, malignancy, active tuberculosis, or hematologic disease were excluded from the study. Thrombocytopenia was detected in 2%, and thrombocytosis was found in 8% of the patients on ED admission. No significant difference was found between the vital signs of patients with normal, low, and high platelet counts. In thrombocytopenic patients, ICU admission (p = 0.011), need for invasive mechanical ventilation (p < 0.001), severe pneumonia (p < 0.001), severe sepsis (p < 0.001) and septic shock (p = 0.009) compared to patients with normal platelet count or thrombocytosis were significantly increased. Patients with thrombocytopenia or thrombocytosis had higher mortality (p = 0.001) and re-admissions (p = 0.011) rates than patients with normal platelet counts. Multivariate logistic regression analysis revealed that thrombocytosis, but not thrombocytopenia, is an independent risk factor for 30 days mortality (OR, 2.720; 95% CI, 1.589–4.657; p < 0.001).

Camon et al. investigated laboratory parameters and mortality in pneumonia patients developing in HIV-infected patients [6]. In this prospective study of 160 patients, platelet count was lower in nonsurvivors (112.7 ± 57.6 vs. 196.6 ± 102.6; p < 0.009).

In the four studies cited above [3–6], only admission thrombocyte counts of the patients were evaluated. Gorelik et al. studied the relationship between mortality and platelet changes during hospitalization in 976 adult community-acquired pneumonia patients [7]. In this study, 90-day mortality was 40.3% in patients with more than 50,000 mm^3^ reductions in platelet counts, 12.3% in those with less than 50,000 mm^3^ changes (described as stable) in platelet counts, and 4.9% in patients with more than 50,000 increase in platelet counts. Additionally, more than a 100,000 increase in platelet counts were associated with lower mortality, and the relative risk for mortality was 0.73 (p < 0.001, 95%, CI 0.64–0.83). Mortality rates were similar among patients with thrombocytopenia, normal platelet count, and thrombocytosis on admission (p = 0.6). In our study, although there was no statistical difference between thrombocytopenia during the follow-up and mortality, it was related to the complicated clinical course. Here we found that platelet counts at the time of admission are not predictors of mortality. Therefore, changes in follow-up rather than platelet measurements at the time of admission should be considered when predicting prognosis in patients with pneumonia.

## 5. Limitations

This is a single-center study. Our ED is located near several nursing home facilities, and the geriatric population is more prevalent in İzmir city than in other locations of Turkey. Most of the patients admitted to ED were elderly and mostly immobilized with additional diseases requiring the need for intensive care. For these reasons, most of the patients in this study have had severe pneumonia (PSI IV and V). Additionally, the low number of thrombocytopenic patients might have led to the detection of no statistical difference in 30 days of mortality.

## 6. Conclusion

Pneumonia constitutes an important part of emergency department visits and in-hospital mortality. However, it was shown that thrombocytopenia and thrombocytosis were associated with in-hospital mortality, platelet count does not account for the clinical scores predicting mortality in patients with pneumonia.

Neither thrombocytopenia nor thrombocytosis is not associated with 30-day mortality. However, patients who developed thrombocytopenia during follow-up needed more intensive care unit admissions, invasive/noninvasive mechanical ventilation, and vasopressor treatment.

Development of thrombocytopenia during follow-up should be considered as a high-risk factor for mortality and complicated clinical course. Early admission to intensive care units or more aggressive treatment should be considered in those patients.

## Figures and Tables

**Table 1:** Demographic and clinical findings of the patients.

*Comorbid diseases and conditions*, n, (%)	
Hypertension	205, (50.6)
Diabetes	121, (29.9)
COPD	115, (28.4)
Immobilization	110, (27.2)
Heart failure	75, (18.5)
Coronary artery disease	72, (17.8)
Nursing home residents	72, (17.8)
Chronic renal failure	48, (11.9)
Stroke	45, (11.1)
Lung malignancy	31, (7.7)
*Vital signs on admission*, (median IQR)	
Systolic blood pressure (mmHg)	120, (104–142)
Diastolic blood pressure (mmHg)	74, (64–82)
Heart rate (beat/min)	100, (85–1169
Respiratory rate (breath/min)	24, (20–28)
Oxygen saturation (%)	90, (84–94)
*Diagnostic imaging*, %, (n)	
Chest X-ray	75, (18.5)
Thorax CT	134, (33.1)
Chest X ray + thorax CT	196, (48.4)
*Findings of diagnostic imaging*, n, (%)	
Multilobar infiltrations	203, (50.1)
Lobar infiltration	169, (41.7)
Patchy infiltration	33, (8.1)
Pleural effusion	167, (41.2)
*PSI score*, n, (%)	
Class I	7, (1.7)
Class II	11, (2.7)
Class III	68, (16.8)
Class IV	139, (34.3)
Class V	180, (44.4)
*30 days mortality and complicated clinical course*, n, (%)	
Mortality	118, (29.1)
ICU admission	169, (41.7)
Invasive mechanical ventilation	105, (25.9)
Noninvasive mechanical ventilation	99, (24.4)
Vasopressor requirement	75, (18.5)

**Table 2:** The relationship between platelet count on admission and mortality-complicated clinical course, initial vital parameters, and severity scores among patient groups.

	Normal thrombocyte (n = 331)	Thrombocytosis (n = 57)	Thrombocytopenia (n = 17)
	n (%)	n (%), p*	n (%) - p**
Mortality (30 days)	96 (29)	16 (28.1), 0.886	6 (35.3), 0.578
Complicated clinical course			
• ICU admission	134 (40.5)	25 (43.9), 0.632	10 (58.8), 0.134
• Invasive mechanical ventilation	81 (24.5)	16 (28.1), 0.562	8 (47.1), 0.037
• Vasopressor	58 (17.5)	13 (22.8), 0.341	4 (23.5), 0.518
• Noninvasive mechanical ventilation	82 (24.8)	11 (19.3), 0.371	6 (35.3), 0.330
Vital parameters	Median (IQR)	Median (IQR), p*	Median (IQR), p**
• Systolic blood pressure (mmHg)	120 (105–145)	120 (97.5–129.5), 0.126	110 (89.5–120), 0.007
• Diastolic blood pressure (mmHg)	75 (64–75)	71 (54.5–81), 0.224	66 (56.5–75.5), 0.018
• Heart rate (beat/min)	99 (84–114)	104 (90.5–120), 0.105	106 (91–125.5), 0.310
• Respiratory rate (breath/min)	24 (20v28)	22 (20–28), 0.329	24 (20–28), 0.691
• Temperature (°C)	36.8 (36–37.9)	37 (36.2–38), 0.449	37 (36.4–37.7), 0.446
• Oxygen saturation (%)	90 (84–93)	91 (83–94.5), 0.564	91 (80–94.5), 0.998
Severity Scores	Median (IQR)	Median (IQR), p*	Median (IQR), p**
• PSI	123 (95–152)	124 (104.5–151), 0.913	154 (105–170.5), 0.05
• CUR–65	2 (1–3)	2 (1.5–3), 0.778	3 (2–4), 0.033

Chi square test was used to analyze the categorical values. Wilcoxon test was used to analyze the numerical values.

* Patients with normal thrombocyte count vs patients with thrombocytosis.

** Patients with normal thrombocyte count vs patients with thrombocytopenia.

**Table 3:** Relationship between platelet count during hospital follow-up and 30 days’ mortality and complicated clinical course among patient groups.

	Normal thrombocyte (n = 152)	Thrombocytosis (n = 68)	Thrombocytopenia (n = 25)
	n, (%)	n, (%), p *	n, (%), p**
Mortality (30 days)	45, (29.6)	20 (29.4), 0.977	11 (44), 0.152
ICU admission	63, (41.4)	33 (48.5), 0.328	16 (64), 0.036
Invasive mechanical ventilation	37, (24.3)	26 (38.2), 0.035	14 (56), 0.001
Vasopressor requirement	29, (19.1)	18 (26.5), 0.216	13 (52), <0.001
Noninvasive mechanical ventilation	39, (25.7)	24 (35.3), 0.144	12 (48), 0.022

Chi square test used to analyze variables.

* Patients with normal thrombocyte count vs patients with thrombocytosis.

** Patients with normal thrombocyte count vs patients with thrombocytopenia.
